# Diagnostic Accuracy of High-Resolution Computed Tomography of Chest in Diagnosing Sputum Smear Positive and Sputum Smear Negative Pulmonary Tuberculosis

**DOI:** 10.7759/cureus.8467

**Published:** 2020-06-05

**Authors:** Waqas Rasheed, Ruby Qureshi, Naila Jabeen, Hyder A Shah, Rashid Naseem Khan

**Affiliations:** 1 Pulmonology, Liaquat College of Medicine & Dentistry, Darul Sehat Hospital, Karachi, PAK; 2 Radiology, South City Hospital, Karachi, PAK; 3 Radiology, Dow University of Health Sciences, Karachi, PAK; 4 Internal Medicine, Liaquat College of Medicine & Dentistry, Darul Sehat Hospital, Karachi, PAK

**Keywords:** sputum smear positive, pulmonary tuberculosis, sputum smear negative, high resolution computed tomography

## Abstract

Introduction

Pulmonary tuberculosis (PTB) is caused by species of organisms in the Mycobacterium tuberculosis complex. It is a major public health problem worldwide and is endemic in Pakistan. Various clinical and biochemical markers exist for its diagnosis. Radiology has an important role in the diagnosis of PTB. Initially, a chest radiograph is warranted for PTB evaluation. High-resolution computed tomography (HRCT) also has high sensitivity and specificity for PTB diagnosis. Features of primary TB include consolidation, lymphadenopathy, pleural effusion and miliary nodules whereas post-primary TB include apical consolidation, nodules and cavitation. The aim of this study was to determine the diagnostic accuracy of HRCT chest in diagnosing sputum smear positive and smear negative PTB.

Methods

A cross-sectional study was conducted at a large tertiary care teaching hospital. A retrospective review of medical records of patients who underwent HRCT chest and sputum acid-fast bacillus (AFB) direct smear and AFB culture for suspicion of PTB was undertaken. All HRCT chest examinations were performed on multislice computed tomography (CT) scanner. On HRCT, PTB was defined as the presence of consolidation, centrilobular nodules, branching nodules with tree in bud appearance with or without lymphadenopathy and pleural effusion. Diagnostic accuracy of HRCT including sensitivity, specificity, positive and negative predictive values was calculated using 2 x 2 table, taking findings of AFB culture as a gold standard.

Results

A total of 108 patients were included in this study with a mean age of 51.85 ± 16.86 years. Diagnostic accuracy of HRCT in diagnosing PTB was found to be 84.26% with sensitivity, specificity, positive predictive value (PPV) and negative predictive value (NPV) of 89.09%, 79.25%, 81.67%, and 87.50%, respectively. In sputum smear positive patients, HRCT has diagnostic accuracy, sensitivity, specificity, PPV and NPV of 87.50%, 88.57%, 84.62%, 93.94%, and 73.33%, respectively. In sputum smear negative patients, HRCT has diagnostic accuracy, sensitivity, specificity, PPV and NPV of 81.67%, 90.00%, 77.50%, 66.67%, and 93.94%, respectively.

Conclusion

HRCT has high sensitivity in diagnosing sputum smear positive and sputum smear negative PTB. The specificity of HRCT in diagnosing sputum smear positive PTB was high, whereas it was slightly low in diagnosing sputum smear negative PTB. Overall diagnostic accuracy of HRCT was high in diagnosing PTB.

## Introduction

Tuberculosis (TB) is the infection caused by species of organisms in Mycobacterium tuberculosis complex. Mycobacterium tuberculosis is the major cause followed by other mycobacteria. However, infection is usually not seen in all the exposed persons. Pulmonary tuberculosis (PTB) is a major public health problem as it affects almost one-third of the population of the world [1]. Pakistan is a developing country where tuberculosis is endemic. In developed countries, immigrants from endemic regions are responsible for increasing PTB incidence [2]. Active PTB usually presents with a cough, which is productive with or without hemoptysis, loss of weight, fever with night sweats, and generalized weakness [3].

Various tests have been developed for PTB diagnosis. In areas with limited resources, in-house real-time polymerase chain reaction (PCR) could be employed with high specificity and sensitivity [4]. C reactive protein (CRP) is a marker of inflammation and carries high sensitivity for screening active PTB [5]. The Xpert mycobacterium tuberculosis/rifampicin (Xpert MTB/RIF) is a World Health Organization (WHO) approved test for the detection of tuberculosis as well as rifampicin resistance and carries high sensitivity and specificity for diagnosing pulmonary and extra-PTB [6]. It also has a high sensitivity in diagnosing PTB in children [7]. Recently Xpert MTB/RIF Ultra has been introduced and has shown promising sensitivity for diagnosing PTB [8].

An important role of radiological imaging exists in the evaluation of PTB. Initially, a chest radiograph is warranted for the evaluation of PTB. Primary tuberculosis manifests as consolidation, enlarged lymph nodes, pleural effusion and miliary nodules on imaging [9]. Apical consolidation, nodules and cavitation are usually seen in post-primary tuberculosis [3]. It has been suggested that adults in developed countries usually develop primary tuberculosis and in adults of PTB endemic countries, post-primary tuberculosis may occur in response to infection by a second mycobacterium strain [3,10]. According to a study, high-resolution computed tomography (HRCT) chest in smear positive patients has a sensitivity and specificity of 90.9% and 96.4%, respectively, in diagnosing active PTB [11]. It is important to utilize accurate means for diagnosing PTB. Therefore, this study was conducted with the aim to determine the diagnostic accuracy of HRCT chest in diagnosing sputum smear positive and smear negative PTB, taking findings of acid-fast bacillus (AFB) culture as the gold standard.

## Materials and methods

This was a cross-sectional study conducted at a tertiary care teaching hospital. A retrospective review of medical records of patients who underwent HRCT chest and sputum for AFB direct smear and AFB culture for suspicion of PTB was undertaken from January 2018 till December 2019. Both adult male and female patients suspected to have PTB were included. Patients were suspected of having tuberculosis if they presented with productive cough with or without hemoptysis, shortness of breath, fever with night sweats or weight loss. Patients on anti-tuberculous therapy (ATT) were excluded. Patients were also excluded if they were not able to produce sputum. Optimal volume of 2 ml was considered adequate for the sputum smear test.

On HRCT, tuberculosis was defined as the presence of consolidation, centrilobular nodules, branching nodules with tree in bud appearance with or without lymphadenopathy, and pleural effusion. AFB culture was performed on Löwenstein-Jensen (LJ) medium. If there was a growth of mycobacteria then the result was positive and if no growth was seen then the result was negative. Statistical Package for Social Sciences (SPSS) version 20.0 (IBM Corp., Armonk, NY) was used for data entry and analysis. Continuous variables such as age were mentioned as mean and standard deviation. Categorical variables like gender, findings of sputum smear test, HRCT, and AFB culture were mentioned as frequency and percentage. Diagnostic accuracy of HRCT including sensitivity, specificity, positive predictive value (PPV) and negative predictive value (NPV) was calculated using 2 x 2 table taking findings of AFB culture as the gold standard. Stratification was done on the basis of sputum smear result and after stratification sensitivity, specificity, PPV, NPV and diagnostic accuracy were calculated.

## Results

A total of 108 patients were included in this study with a mean age of 51.85 ± 16.86 years. Out of 108 patients, a total of 64 (59.3%) were males and 44 (40.7%) were females. A total of 60 (55.6%) patients had sputum smear negative and 48 (44.4%) had sputum smear positive. These characteristics are summarized in Table 1.

**Table 1:** Baseline characteristics of the patients ^ǂ^Mean ± SD, n: number

Baseline characteristics of the patients (n = 108)		
	n	%
Age, years	51.85 ± 16.86^ǂ^	
Gender		
Males	64	59.3
Females	44	40.7
Sputum smear test		
Positive	48	44.4
Negative	60	55.6

Among 108 patients, positive findings on HRCT were seen in 60 (55.6%) patients and positive findings on AFB culture were seen in 55 (50.9%) patients (Table 2).

**Table 2:** HRCT chest and AFB culture findings HRCT, high-resolution computed tomography; AFB, acid-fast bacillus

HRCT chest and AFB culture findings (n = 108)			
HRCT chest findings	AFB culture findings		Total
	Positive	Negative	
Positive	49	11	60
Negative	6	42	48
Total	55	53	108

Diagnostic accuracy of HRCT in diagnosing PTB was found to be 84.26% with sensitivity, specificity, PPV and NPV of 89.09%, 79.25%, 81.67%, and 87.50%, respectively. In sputum smear positive patients, HRCT had diagnostic accuracy, sensitivity, specificity, PPV and NPV of 87.50%, 88.57%, 84.62%, 93.94% and 73.33%, respectively. In sputum smear negative patients, HRCT had diagnostic accuracy, sensitivity, specificity, PPV and NPV of 81.67%, 90.00%, 77.50%, 66.67%, and 93.94%, respectively.

## Discussion

Any organ system of the body can be affected by tuberculosis and lungs are the most common site usually involved. Sputum smear is often used initially for PTB evaluation. Sputum smear results may take several days to come and the results of AFB culture may take several weeks [12]. Therefore, imaging has an important role in PTB evaluation. In addition to diagnosis, imaging may evaluate treatment response and may also detect any complications associated with this illness [12].

PTB is differentiated into primary and post-primary TB, having different radiological features with slight overlapping. Primary TB may infect lung parenchyma, lymph nodes, pleura or tracheobronchial tree. The focus of primary TB within parenchyma is Ghon focus and together with enlarged lymph node constitutes Ghon/Ranke complex [13]. Post-primary PTB occurs in previously infected/sensitized patients via re-infection/re-activation and this may be due to malnutrition or immunosuppression [12].

PTB is a curable disease, which is endemic in Pakistan. The gold standard for PTB diagnosis is AFB culture. One potential diagnostic challenge is also sputum smear negativity with a suspicion of PTB. Various biochemical tests are available that are usually not widely available. Therefore, HRCT can aid in diagnosis and initiating empirical treatment. Moreover, a normal study can help in the exclusion of disease process.

The present study was conducted to evaluate diagnostic accuracy, including sensitivity, specificity, PPV, and NPV of HRCT chest in diagnosing sputum smear positive and smear negative PTB. The study excluded patients undergoing anti-tuberculous treatment. Findings of AFB culture were taken as the gold standard.

Our study has shown that HRCT has a sensitivity of 88.57% and 90.00% in diagnosing sputum smear positive and smear negative PTB. Previous studies have shown a high sensitivity of HRCT in diagnosing PTB. A previous study has shown a sensitivity of 96.4% in diagnosing smear positive active PTB, which was higher as compared to our study [11]. Another study has shown the sensitivity of 82.7% in diagnosing smear negative PTB, which was slightly lower than the one as compared to our study [14]. Epidemiological differences in PTB prevalence and risk factors could be a possible factor in this difference of sensitivities. Moreover, another potential reason could be a difference in scanning parameters of HRCT.

According to the results of our study, HRCT has a high specificity of 84.62% for diagnosing sputum smear positive PTB. Whereas, the specificity for detecting sputum smear negative PTB was 77.50%. A previous study has shown a high specificity of 90% in diagnosing PTB [15]. Another study showed that HRCT has a specificity of 70.5% for diagnosing smear negative PTB, which was lower than the one reported in our study [16]. For sputum smear positive PTB, a study has shown a higher specificity as compared to our study for diagnosis [11].

HRCT has shown a high NPV for diagnosing sputum smear positive and smear negative PTB. According to our study, PPV of HRCT for diagnosing sputum smear positive PTB was high, whereas it was low for diagnosing sputum smear negative PTB.

An alternative to HRCT is MRI and it is being increasingly studied as a radiation-free tool for diagnosing PTB. A study was conducted on 50 PTB patients that were proven by culture. Both HRCT and MRI were studied. The study results showed that both MRI and HRCT identified pulmonary abnormalities associated with tuberculosis in all the patients [17]. Moreover, MRI had a high resolution that identified caseation, nodal, pleural or parenchymal inhomogeneity better than HRCT [17].

Studies have also shown that radiological manifestation combined with serological testing can help improve diagnosis of PTB. Authors from a previous study [16] evaluated HRCT and interferon-gamma releasing assay (IGRA) for sputum smear negative PTB. The study concluded that combined HRCT and IGRA improve the detection of PTB in cases of sputum smear negativity and help in management by the initiation of treatment early [16].

A study was carried out in the population of Uganda regarding the use of Gene Xpert and chest radiograph in patients with smear negative PTB. The study was carried out on 123 adult patients. The study concluded that a single Xpert test did not yield a positive result in the majority of sputum smear negative PTB. Moreover, chest radiograph over-estimated the PTB in smear negative cases [18].

A potential limitation of this study was its small sample size. Moreover, another limitation of this study was that it was carried out in a population of PTB endemic countries. It is recommended that further studies of multicentric nature should be carried out on a larger sample size to validate the results of this study.

## Conclusions

HRCT has high sensitivity in diagnosing sputum smear positive and sputum smear negative PTB. The specificity of HRCT in diagnosing sputum smear positive PTB was high, whereas it was slightly low in diagnosing sputum smear negative PTB. Overall diagnostic accuracy of HRCT was high in PTB diagnosis. HRCT had low PPV in diagnosing sputum smear negative PTB, whereas high PPV in diagnosing sputum smear positive PTB.
